# Direct detection of *Helicobacter pylori* from biopsies of patients in Lagos, Nigeria using real-time PCR—a pilot study

**DOI:** 10.1186/s13104-021-05505-y

**Published:** 2021-03-09

**Authors:** A. Ajayi, T. Jolaiya, S. I. Smith

**Affiliations:** 1 Department of Molecular Biology and Biotechnology, Nigerian Institute of Medical Research, Yaba, Lagos, Nigeria; 2 Department of Microbiology, University of Lagos, Lagos, Nigeria; 3 Mountain Top University, Makogi Oba, Ogun Nigeria

**Keywords:** *Helicobacter pylori*, Diagnosis, Real-time PCR, Biopsy, Ulcer

## Abstract

**Objective:**

Prompt diagnosis of *Helicobacter pylori* infection is essential for proper treatment and eradication of the pathogen because prolonged infection could lead to gastric cancer. Sensitive and cost effective diagnostic methods are key to guiding treatment options that will reduce mortality. This study was aimed at detecting *H. pylori* from biopsies of peptic ulcer patients. Real-time PCR using TaqMan and EvaGreen assays targeting 16S rRNA and *ureA* genes were used to detect *H. pylori* DNA extracted from 40 biopsy samples comprising 20 biopsies obtained from the antrum and 20 from the corpus of 20 patients undergoing endoscopy for duodenal ulcer investigation in Lagos, Nigeria.

**Results:**

*H. pylori* was detected in 80% of the biopsy samples by combined cycle threshold (*C*_*t*_) and melting temperature (*T*_*m*_) values. Mean *C*_*t*_ value for *ureA* gene ranged from 21.40 to 37.53 and 22.71 to 35.44 for *16SrRNA* gene. Average melting temperatures (*T*_*m*_) of 81.57 and 82.90 °C among amplicons of *ureA* and 16S rRNA were observed respectively. *H. pylori* DNA was generally detected in biopsies collected from antrum and corpus. Real-time PCR in the diagnosis of *H. pylori* can be considered a simple, low cost and efficient alternative or addition to the gold standard.

**Supplementary Information:**

The online version contains supplementary material available at 10.1186/s13104-021-05505-y.

## Introduction

Gastritis, duodenal ulcer, gastric ulcer and in some cases gastric cancers are hallmarks of *Helicobacter pylori* infection. *H. pylori* is a Gram negative bacterium that colonize 50% of the stomach of humans globally [1, 2]. *H. pylori* possess several virulence factors including the production of urease that enable it successfully colonize the stomach where it can persist for a long period of time. The pathogen has been classified as a type 1 carcinogen hence, its persistence in infection without eradication may lead to chronic gastritis, mucosa-associated lymphoid tissue (MALT) lymphoma and gastric cancer [3–5]. *H. pylori* is a fastidious bacterium making it very delicate to culture. Although non-invasive methods of detecting *H. pylori* exist [6], culture remains the gold standard which requires competence and a lot of materials making it expensive. Detection of *H. pylori* DNA directly from biopsies by molecular methods especially PCR have been reported by several workers [7, 8] with excellent sensitivity and specificity. In Nigeria, the use of conventional PCR in the detection of *H. pylori* DNA from biopsies have been demonstrated [9, 10]. However, the use of real-time PCR in the detection of *H. pylori* in biopsies have not been explored in the country. The purpose of this study was to detect *H. pylori* DNA isolated from gastric biopsies (corpus and antrum) obtained from patients in Lagos Nigeria.

## Main text

Samples used for this study were biopsies obtained from 20 patients undergoing endoscopy for duodenal ulcer investigation in Lagos, Nigeria. A total of 40 biopsies comprising 20 obtained from the corpus and 20 from the antrum were analysed.

## Methodology

DNA was extracted from gastric biopsies (corpus and antrum) and *H. pylori* reference strain J99 (NCBI:txid85963) with QIAamp DNA Mini Kit (Qiagen, Hilden, Germany) according to manufacturer’s instructions.

Detection of *H. pylori* was done by singleplex real-time PCR amplifying fragments of 16S rRNA and *ureA* genes using specific primers and probes listed in Additional file 1: Table S1 with slight modification (quencher of probes was Black Hole Quencher (BHQ) instead of TAMARA). Detection of *H. pylori* targeting the two set of primers was first validated by using *H. pylori* DNA extracted from reference *H. pylori* strain J99 (NCBI:txid85963) and DNA extracted from *Salmonella* Typhimurium ATCC 14028, *Staphylococcus aureus* ATCC 29213, *Pseudomonas aeruginosa* ATCC 27853 and *Escherichia coli* ATCC 29522. A standard curve of the assay was determined as serial dilution of DNA from *H. pylori* J99 positive control which was prepared with final concentration of 10^1^–10^5^; reactions were ran in triplicate. The curve was determined by plotting threshold cycle (Ct) value against count of log DNA copies from which positive samples were considered as amplification plot with Ct values < 40. Real-time PCR was carried out in a StepOne Real-Time PCR system (Applied Biosystem, Singapore). A 20 µL reaction was used in both the TaqMan and EvaGreen assays which contained 11.2 µL nuclease free water, 4 µL of Solis Biodyne 5 × HOT FIREPol® Probe qPCR Mix Plus (for TaqMan assay) and 5X HOT FIREPol® EvaGreen Supermix (Solis Biodyne Tartu, Estonia), 0.4 µL (10 µM) each of both forward and reverse primers and 4 µL of (20 ng/µL) DNA template. PCR cycling parameters for the EvaGreen assay were an initial denaturation at 95 °C for 12 min and 40 cycles of denaturation at 95 °C for 15 s, annealing for 1 min at 54 °C for 16S rRNA gene, 55 °C annealing temperature for *ureA* gene*,* extension at 72 °C for 20 s and a melting step. While PCR cycling parameters for the TaqMan assay were initial denaturation at 95 °C for 12 min, denaturation at 95 °C for 15 s, annealing/elongation at 54 °C for 16S rRNA gene and 55 °C for *ureA* gene both for 1 min. After which amplification results were analysed with StepOne Software v.23 and GraphPad Prism software version 5 (GraphPad Software, LA Jolla, CA, USA).

## Results

**Fig. 1:**
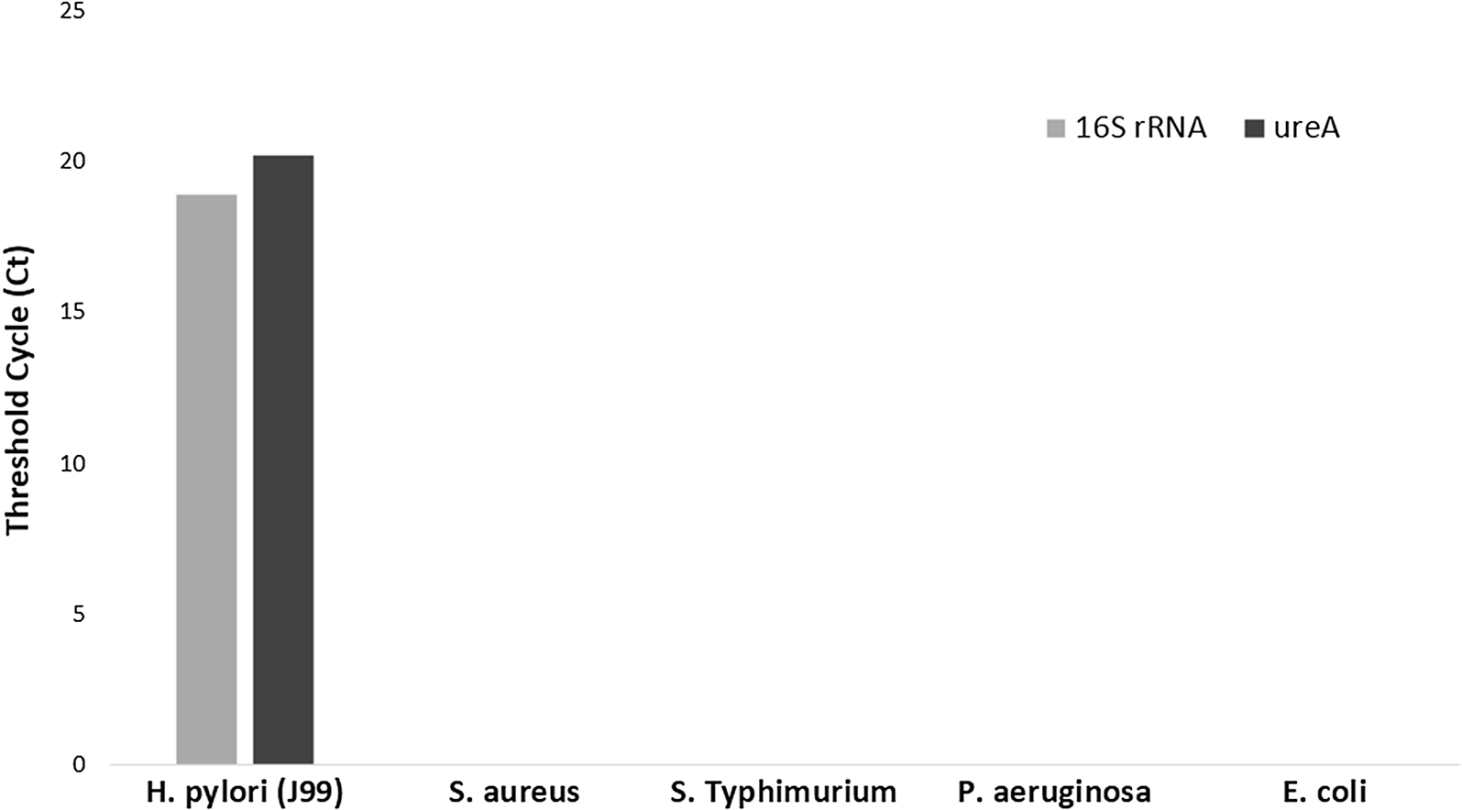
Threshold cycle (*c*_*t*_) value indicated for reference *H. pylori* (J99) strain on the *y-axis*. There was no amplification for other bacterial pathogens including *S. aureus*, S. Typhimurium, *P. aeruginosa* and *E. coli* (*x-axis*) indicating specificity of primer/probe sets targeting 16S rRNA and *ureA* genes of *H. pylori*

Real-time PCR assay for the detection of *H. pylori* from genomic DNA extracted from biopsies and extracted DNA from *H. pylori* reference strain J99 (NCBI:txid85963) showed amplification curves for all targeted genes (*ureA* and 16S rRNA). However, there was no amplification with DNA extracted from other bacterial pathogens (*Salmonella* Typhimurium ATCC 14028, *Staphylococcus aureus* ATCC 29,213, *Pseudomonas aeruginosa* ATCC and *Escherichia coli* ATCC 29522) as shown in Fig. 1 indicating specificity of primers and probes used.

**Fig. 2:**
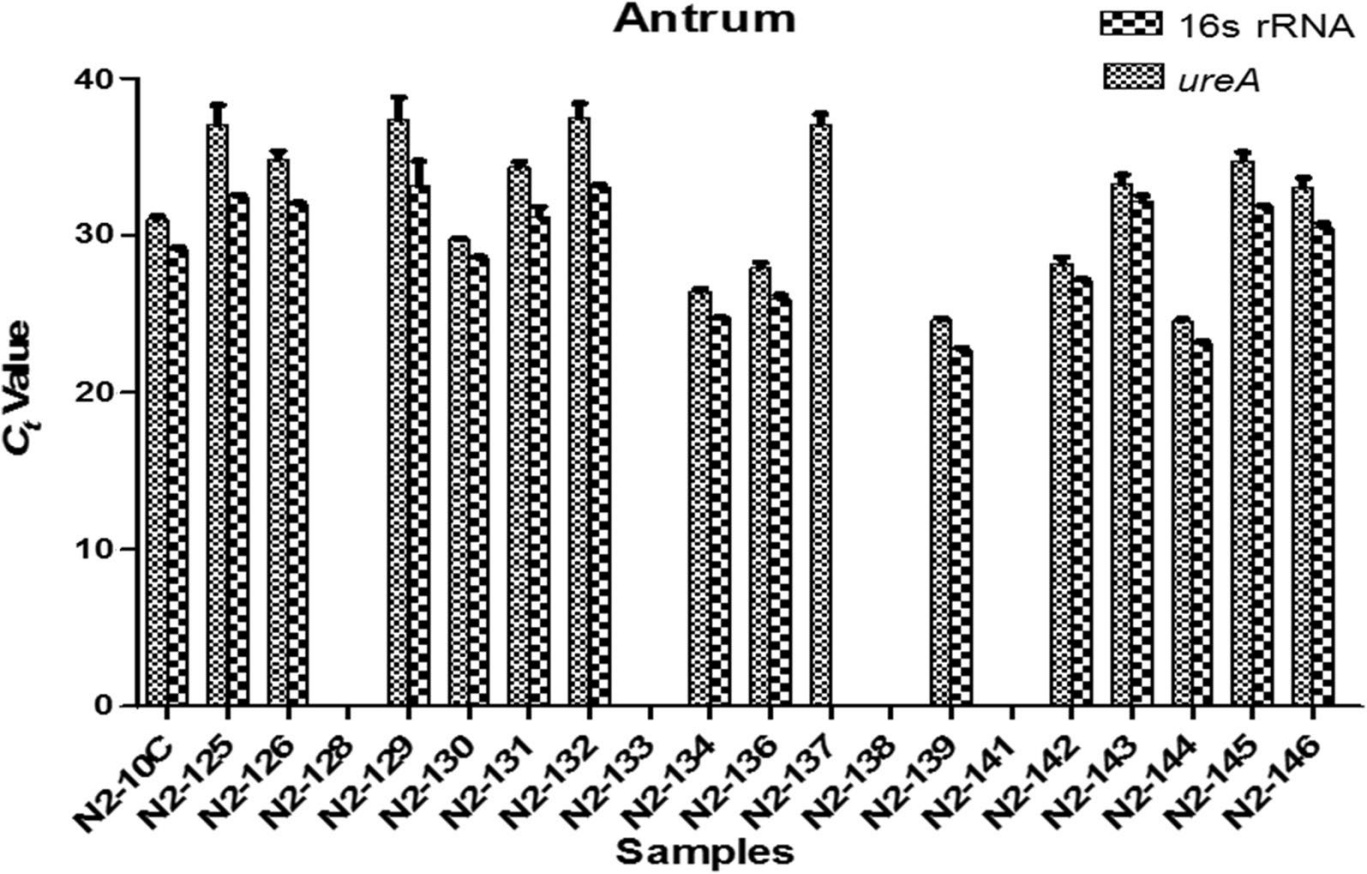
Threshold cycle (*C*_*t*_) values for *ureA* and 16S rRNA gene amplification in antrum biopsy samples is indicated on the *y-axis*. Each value is mean of triplicates. While samples N2-134, N2-136, N2-139 and N2-144 had relatively low *C*_*t*_ values for both genes, there was no amplification in samples N2-128, N2-133, N2-138 and N2-141. Error bar indicates the ± standard deviation (SD)

**Fig. 3:**
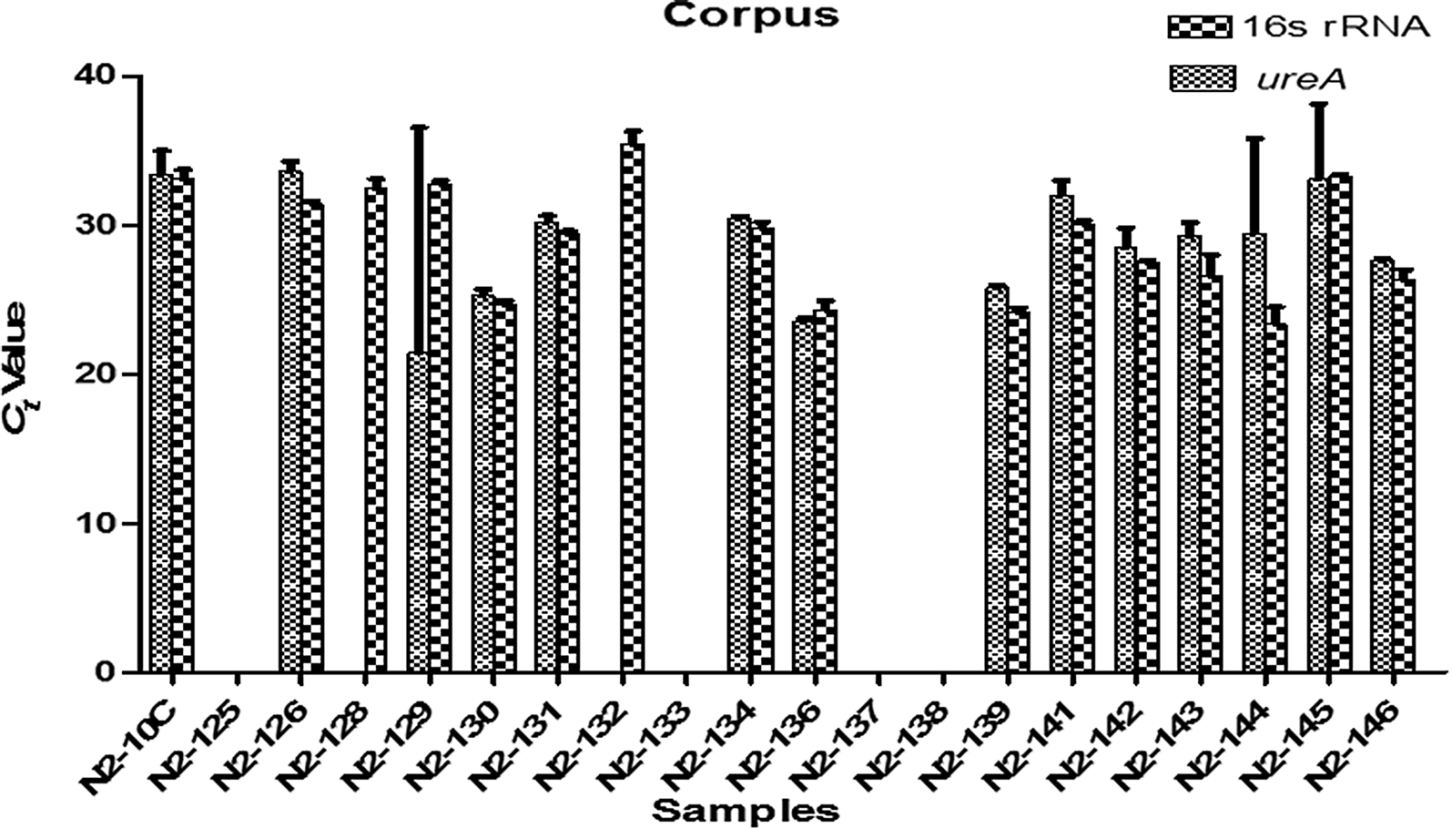
Threshold cycle (*C*_*t*_) values for *ureA* and 16S rRNA genes amplification in corpus biopsy samples. Each value is mean of triplicates. Amplification was not observe in N2-125, N2-133, N2-137 and N2-138. Error bar indicates the ± standard deviation (SD)

Eighty percent of the samples were positive for *H. pylori*. However, *H. pylori* was detected in the antrum of two patients but was not detected in the corpus. The reverse was observed in a third patient in which *H. pylori* was detected in the corpus but absent in the antrum. The logarithm of the number of *H. pylori* DNA copies in the samples correlated largely well with the *ure*A and 16S rRNA threshold cycle (*C*_*t*_) values which is a representation of the DNA copy number in the PCR reaction. Mean *C*_*t*_ value for *ureA* gene ranged from Mean ± SD: 21.40 ± 15.14 to 37.53 ± 0.89 and for 16S rRNA gene Mean ± SD: 22.71 ± 0.12 to 35.44 ± 0.87 as shown in Figs. 2 and 3. Correlation coefficient (*R*^2^) and amplification efficiency (*E*) was 0.994/90.35%, 0.997/97.94% for *ureA* and 16S rRNA respectively.

The intercalating chemistry EvaGreen used showed smooth melting curve (Additional file 2: Figure S1) and average melting temperatures (*T*_*m*_) of 81.57 and 82.90 °C among amplicons of *ureA* and 16S rRNA respectively.

## Discussion

*Helicobacter pylori* infection remains a major public health issue around the world. The prevalence of *H. pylori* infection in Nigeria is estimated at about 87.7% which indicates a high infection burden [1] making prompt, accurate and efficient diagnosis imperative. The PCR results for the detection of *H. pylori* from biopsies and other bacterial pathogens indicated the reliability of using the *H. pylori ureA* and 16S rRNA primer set in this study. Eighty percent of the samples were positive for *H. pylori* with the assay showing great efficiency in detecting small quantities of *H. pylori* DNA. Correlation coefficient (*R*^2^) and amplification efficiency (*E*) of both *ureA* (0.994/90.35%) and 16S rRNA (0.997/97.94%) proved to be good. Hence it could be inferred that these targets had great specificity since all *H. pylori* strains harbour the gene that encodes urease. In the study of Ramı´rez-La´zaro et al. [11], they suggested the combination of both 16S rRNA and *ureA* genes in the diagnosis of *H. pylori* from biopsies for better sensitivity. However, in the study by Beer-Davidson et al. [12] in which they reported the detection of *H. pylori* in stool samples of children in Israel using real-time PCR targeting urease gene, it was observed that the gene gave a clearer amplification curve compared to 16S rRNA gene. In this study, the mean temperature separation between *ureA* (81.57 °C) and 16S rRNA (82.90 °C) was 1.33 °C. Thus it could be asserted that amplification products of *ureA* gene could easily be distinguished from 16S rRNA even in a multiplex qPCR reaction. Contreras et al. [13] reported a melting temperature range between 57.0 and 57.4 °C that enabled them detect 16S rRNA single mutation associated with antibiotic resistance in *H. pylori* strains isolated from biopsies in Venezuela compared to a higher T_m_ that was observed in wild type strains. The detection of *H. pylori* in biopsies collected from the antrum and corpus is widely reported. Pichon et al. [14], reported the detection of *H. pylori* in both biopsies of antrum and corpus obtained from patients with *H. pylori* infection. However, in this present study there was zero detection of *H. pylori* in the corpus of two patients in which *H. pylori* was detected in their antrum biopsy samples. Similarly, in one patient, *H. pylori* was detected in corpus biopsy but absent in the antrum. Palamides et al. [15] reported similar findings in which *H. pylori* was detected in biopsies from either antrum or corpus of some patients using conventional PCR. This suggest that there are variations in the distribution of *H. pylori* in the gut of *H. pylori* infected patients. Lan et al. [16] reported in their study that corpus biopsy enhances the detection of *H. pylori* infection. Similarly, Latif et al. [17] opined that in addition to antral biopsy, corpus biopsy increases the sensitivity in the detection of *H. pylori* infection.

## Conclusion

Real-time PCR in the diagnosis of *H. pylori* can be considered an alternative or in addition to the gold standard and including histology since it relies on the detection of DNA isolated from biopsies and not necessarily viable bacteria coupled with its competitive cost [11]. Furthermore, direct detection of *H. pylori* from biopsies can circumvent the difficulty and extended time lapse encountered with culture.

## Limitation of study

It would be difficult to make far reaching conclusion as the number of samples in this study are few and other diagnostic methods such as histology and culture were not evaluated alongside real-time PCR. Hence, future study should increase the number of samples and evaluate other diagnostic methods alongside.

## Supplementary Information

**Additional file 1: Table S1.** Primers used in the detection of *H. pylori* by TaqMan and EvaGreen qPCR assay.

**Additional file 2: Figure S1.** Melting curve of EvaGreen real-time PCR targeting **a** *16SrRNA* **b** *ureA*. Melting peaks were derived by the plot of derivative reporter (−R) against temperature (^o^C).

## Data Availability

The datasets used and/or analysed during the current study available from the corresponding author on reasonable request.

## Abbreviations

ATCC: American Type Culture Collection
BHQ: Black Hole Quencher
Ct: Cycle threshold
MALT: Mucosa-associated Lymphoid Tissue
Tm: Melting Temperature
