# Supplementary material for: Direct detection of Helicobacter pylori from biopsies of patients in Lagos, Nigeria using real-time PCR—a pilot study
Source: BMC Res Notes. 2021 Mar 9;14:90. doi: 10.1186/s13104-021-05505-y (PMC7941902; doi:10.1186/s13104-021-05505-y)
Supplement: Supplementary file 1 — Additional file 1: Table S1. Primers used in the detection of H. pylori by TaqMan and EvaGreen qPCR assay. [file 13104_2021_5505_MOESM1_ESM.docx]

Table S1. Primers used in the detection of *H. pylori* by TaqMan and EvaGreen qPCR assay

| **Gene** | **Primer** | **Length (bp)** | **Reference** |
| --- | --- | --- | --- |
| 16S rRNA | FW 5’-ctcattgcgaaggcgacct-3’  RV 5’-tctaatcctgtttgctcccca-3’ | 76 | [11] |
| *ureA* | FW 5’-cgtggcaagcatgatccat-3’  RV 5’-gggtatgcacggttacgagttt-3’ | 77 |  |
| *ureA* | 5’-(6-FAM)-tcggaaacatcgcttcaatacccactt-BHQ-3’ |  |  |
| 16S rRNA | 5’-(6-FAM)-attactgagctgattgcgcgaaagc-BHQ-3’ |  |  |
