# Supplementary figures and images for: Direct detection of Helicobacter pylori from biopsies of patients in Lagos, Nigeria using real-time PCR—a pilot study
Source: BMC Res Notes. 2021 Mar 9;14:90. doi: 10.1186/s13104-021-05505-y (PMC7941902; doi:10.1186/s13104-021-05505-y)

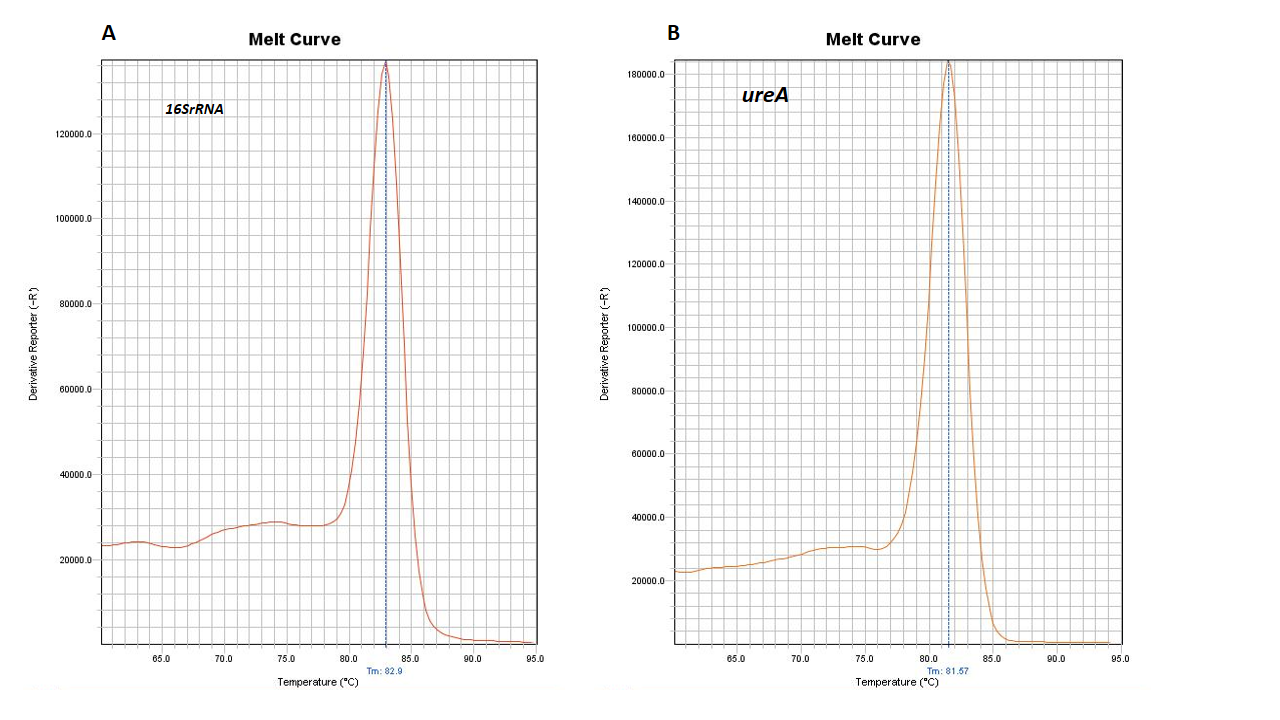

Supplement: Supplementary file 2 — Additional file 2: Figure S1. Melting curve of EvaGreen real-time PCR targeting a 16SrRNA b ureA. Melting peaks were derived by the plot of derivative reporter (−R) against temperature (oC). [file 13104_2021_5505_MOESM2_ESM.tif]
